# Development of selective medium for IMP-type carbapenemase-producing *Enterobacteriaceae* in stool specimens

**DOI:** 10.1186/s12879-017-2312-1

**Published:** 2017-03-24

**Authors:** Norihisa Yamamoto, Ryuji Kawahara, Yukihiro Akeda, Rathina Kumar Shanmugakani, Hisao Yoshida, Hideharu Hagiya, Naohiro Hara, Isao Nishi, Satomi Yukawa, Rumiko Asada, Yumi Sasaki, Kazuhiro Maeda, Noriko Sakamoto, Shigeyuki Hamada, Kazunori Tomono

**Affiliations:** 1 Department of Infection Control and Prevention, Osaka University Graduate School of Medicine, 2-2 Yamadaoka, Suita, Osaka 565-0871 Japan; 2 Research Institute for Microbial Diseases, Osaka University, Osaka, Japan; 3 Department of Bacteriology, Osaka Prefectural Institute of Public Health, Osaka, Japan; 4 Laboratory for Clinical Investigation, Osaka University Hospital, Osaka, Japan; 5 Ibaraki Public Health Center, Osaka, Japan; 6 Clinical Laboratory Department Section 1, The Research Foundation for Microbial Diseases of Osaka University, Osaka, Japan

**Keywords:** Carbapenemase-producing *Enterobacteriaceae*, IMP, Selective culture medium, Faecal specimens

## Abstract

**Background:**

Identification of carbapenemase-producing *Enterobacteriaceae* (CPE) in faecal specimens is challenging. This fact is particularly critical because low-level carbapenem-resistant organisms such as IMP-producing CPE are most prevalent in Japan. We developed a modified selective medium more suitable for IMP-type CPE.

**Methods:**

Fifteen reference CPE strains producing different types of β-lactamases were used to evaluate the commercially available CHROMagar KPC and chromID CARBA as well as the newly prepared MC-ECC medium (CHROMagar ECC supplemented with meropenem, cloxacillin, and ZnSO_4_) and M-ECC medium (CHROMagar ECC supplemented with meropenem and ZnSO_4_). A total of 1035 clinical samples were then examined to detect CPE using chromID CARBA and M-ECC medium.

**Results:**

All tested strains producing NDM-, KPC-, and OXA-48-carbapenemases were successfully cultured in the media employed. Although most of the IMP-positive strains did not grow in CHROMagar KPC, chromID CARBA, or MC-ECC, all tested strains grew on M-ECC. When faecal samples were applied to the media, M-ECC medium allowed the best growth of IMP-type CPE with a significantly higher sensitivity (99.3%) than that of chromID CARBA (13.9%).

**Conclusions:**

M-ECC medium was determined as the most favourable selective medium for the detection of IMP-type CPE as well as other types of CPE.

**Electronic supplementary material:**

The online version of this article (doi:10.1186/s12879-017-2312-1) contains supplementary material, which is available to authorized users.

## Background

Infection with carbapenemase-producing *Enterobacteriaceae* (CPE) has been associated with high rates of morbidity and mortality [1, 2]. Regular surveillance of CPE is critically important to prevent the spread of these pathogens in healthcare settings [3]. The main reservoir of CPE is the intestinal tract, and faecal specimens are conventionally used for the screening of CPE. However, isolation of CPE from faecal specimens is difficult because CPE usually exists as a small proportion of the overall bacterial load [4, 5]. Furthermore, among several types of carbapenemases, enzymes such as OXA-48 and IMP have low capability of hydrolysing carbapenems [6]. These data indicate the difficulties in screening for CPE in stool specimens [3–5, 7].

Several phenotypic methods for CPE screening have been evaluated, but no standardised method has been established to date [4, 5]. Even highly carbapenem-resistant phenotypes such as NDM-type CPE are difficult to identify if cultured as a small inoculum in selective medium [8]. A similar phenomenon is observed when screening for vancomycin-resistant *Enterococcus* [9]. The sensitivity of screening largely depends on the minimum inhibitory concentration (MIC) of the drug for the isolate and the bacterial load. This phenomenon is especially important in Japan because the most prevalent CPE in Japan are IMP-1 and IMP-6- types, which show low-level resistance to carbapenems. Furthermore, IMP-6-type isolates are generally susceptible to imipenem, but resistant to meropenem [10, 11]. Therefore, IMP-type CPE is occasionally misidentified in laboratory examinations and is referred to as ‘stealth-type CPE’ to draw attention to this type of CPE [11]. Several reports indicated difficulties in screening for OXA-48-type isolates, which are widely known to display low-level resistance [12]. Meanwhile, a reliable phenotypic method for IMP-type CPE has not been investigated [4]. In this study, we developed a selective medium for IMP-type CPE.

## Methods

### Bacterial isolates, clinical specimens, and ethical statements

Four selective culture media (*vide infra*) were examined with bacterial isolates as well as clinical faecal specimens. Fifteen CPE isolates of *Enterobacteriaceae* and five non-CPE isolates were used (Table 1). The MICs of meropenem and imipenem for each isolate were determined using Etest strips (bioMérieux Clinical Diagnostics, Marcy l’Etoile, France), and the genotypic characteristics of each isolate with respect to carbapenemase and extended-spectrum beta-lactamase (ESBL) genes were determined via PCR as previously described [13].

**Table 1:** Properties of the bacterial strains used and average recoveries of CFU on each selective agar medium

Strain	Species	Type of β-lactamase		MIC (mg/L)		% CFU compared to that of each strain grown in MHA			
		Carbapenemase	ESBL	MEPM	IPM	CHROMagar KPC	chromID CARBA	MC-ECC	M-ECC
1504-16	*Escherichia coli*	IMP-6	CTX-M-15, CTX-M-2	8	0.5	0*	0*	91	97
1502-08	*E. coli*	IMP-6	-	8	0.5	0*	0*	0*	42*
1405-06	*E. coli*	IMP-6	CTX-M-15, CTX-M-2	3	0.38	0*	0*	74	91
1502-25	*E. coli*	IMP-6	CTX-M-2	3	3	44*	112	80	98
1409-10	*Klebsiella pneumoniae*	IMP-6	CTX-M-2	1	0.19	0*	0*	4*	95
1502-02	*K. pneumoniae*	IMP-6	-	4	0.25	0*	0*	3*	123
1409-09	*K. pneumoniae*	IMP-6	CTX-M-2	0.5	0.25	0*	0*	0*	27*
1212-13	*K. pneumoniae*	IMP-6	CTX-M-2	0.5	0.25	0*	0*	8*	66
1412-36	*K. pneumoniae*	IMP-1	CTX-M-2	3	0.5	0*	0*	33*	97
1502-06	*K. pneumoniae*	IMP-1	-	1	1	0*	114	75	74
1011-12	*K. pneumoniae*	KPC-2	-	>32	>32	97	93	93	100
BAA-1705	*K. pneumoniae*	KPC-2	-	32	32	109	115	157	155
BAA-2470	*K. pneumoniae*	NDM-1	CTX-M-15	16	32	41	112	119	116
BAA-2471	*E. coli*	NDM-1	CTX-M-15	>32	>32	138	114	170	149
TRKP-54	*K. pneumoniae*	OXA-48	-	16	32	99	89	105	95
1502-04	*Enterobacter cloacae*	-	-	2	2	10*	0*	0*	21*
1012-01	*Serratia marcescens*	-	-	6	>32	3*	113	127	98
1104-18	*E. cloacae*	-	-	0.19	0.5	0*	0*	0*	0*
1004-112	*E. coli*	-	CTX-M-2	0.016	0.25	0*	0*	0*	0*
1004-25	*K. pneumoniae*	-	CTX-M-2	0.032	0.38	0*	0*	0*	0*

*ESBL* extended-spectrum β-lactamase, *MEPM* meropenem, *IPM* imipenem, *MHA* Mueller-Hinton agar

*p < 0.01

Faecal samples were collected from 30 hospitals in northern Osaka in December 2015 through the regional surveillance project performed by Osaka University, four regional public health centres, and Osaka Prefectural Institute of Public Health. Ethical approval was obtained from the Ethical Review Boards of each institution.

### Evaluation of selective agar using bacterial isolates

Bacteria in the stool of colonised patients are typically present at low abundances [3, 4], although the inoculum size used to determine CLSI susceptibility breakpoints and MICs ranged from 10^4^ cells per spot (agar dilution) to 5 × 10^5^ cells per millilitre (broth microdilution) [14]. Therefore, to simulate experiments using stool samples, we inoculated each plate with approximately 10^2^ CFU and compared the CFU counts in selective agar media as previously described [8]. Overnight cultures of each isolate in brain-heart infusion broth (BD Diagnostic Systems, Detroit, MI, USA) were serially diluted and inoculated in triplicate plates. CFU counts were compared among Mueller-Hinton agar (MHA) (BD Diagnostic Systems) as well as two commercial and two in-house prepared agar media: (1) chromID CARBA (bioMérieux, Paris, France), (2) CHROMagar KPC (CHROMagar Microbiology, Paris, France), (3) MC-ECC medium (CHROMagar ECC [CHROMagar Microbiology] supplemented with 0.25 μg/mL meropenem, 250 μg/mL cloxacillin, and 70 μg/mL ZnSO_4_), and (4) M-ECC medium (CHROMagar ECC supplemented with 0.25 μg/mL meropenem and 70 μg/mL ZnSO_4_). Each plate was incubated at 37 °C for 24 h, and the percent recovery was calculated as the average colony count of a given isolate divided by that in MHA. The difference in the CFU count between MHA and each selective medium was assessed using a two-tailed Student’s *t* test with GraphPad Prism software (GraphPad Software, La Jolla, CA, USA).

### Evaluation of selective agar using stool specimens

Clinical faecal specimens from the patients were collected with Seed Swab No. 1 (Eiken Chemical Co., Ltd., Tokyo, Japan) and stored at 4 °C until examination. The samples were inoculated directly from the swab into chromID CARBA medium and M-ECC medium without pre-incubation. Positive colonies were selected as defined by the manufacturer’s instructions and examined for specification of bacterial isolates and antimicrobial susceptibility using a MicroScan WalkAway Plus system (Beckman Coulter, Brea, CA, USA). The results were interpreted according to the 2012 guidelines (M100-S22) of the Clinical Laboratory Standards Institute (Wayne, PA, USA). When the isolates showed intermediate or resistance to meropenem or imipenem, their carbapenemase genotypes were analysed by PCR as previously described [13]. An isolate positive for the *bla*_IMP_ gene was further analysed to identify the *bla*_IMP-1_ or *bla*_IMP-6_ type by sequencing the acquired amplified products using a primer set targeting *bla*_IMP_ (IMP-1_469F: TTTATATTTTTGTTTTGCAGCATTGC; IMP-1_1277R: CGCGTTGTGGAATACTTTGC). Sensitivity and specificity of media for *bla*_IMP_-type CPE identification were calculated using GraphPad Prism software.

## Results and discussion

We evaluated three major selective media, the chromID CARBA, CHROMagar KPC, and MC-ECC that was made in reference to SuperCarba medium [15]. The NDM-, KPC-, and OXA-48-positive strains were recovered with both chromID CARBA and CHROMagar KPC, but eight IMP-positive strains were not recovered in any selective media (Table 1). MC-ECC showed better recoveries than chromID CARBA and CHROMagar KPC, although six IMP-positive strains were not recovered. This result is similar to previous reports [7, 16, 17]. IMP-producing CPE was also difficult to detect when a small number was inoculated.

Several reports indicated that commercially available SuperCarba, chromID OXA-48, and chromID ESBL are favourable selective media [2, 12, 15, 18]. Unfortunately, neither SuperCarba nor chromID OXA-48 was available in Japan at the time of our research, and chromID ESBL is not appropriate for CPE detection due to the high prevalence of ESBL in Japan [19]. Therefore, we developed MC-ECC medium as an in-house SuperCarba. Surprisingly, however, a significant reduction in the apparent CFU was observed in six out of the 10 IMP-positive isolates plated on MC-ECC medium. This result could be explained by our supplementary experiment indicating that the MIC of meropenem for IMP-type strains decreases when cloxacillin is added (Additional file 1: Table S1). Therefore, we removed cloxacillin from the MC-ECC medium to increase the sensitivity and referred to the resulting medium as M-ECC. As a result, all CPE strains could be detected in M-ECC medium (Table 1).

In the next series of experiments, a total of 1024 stool samples and 11 rectal swabs were examined to compare the capability of M-ECC and chromID CARBA to detect CPE in stool samples. The average time interval from sample acquisition to examination was 4.66 ± 2.66 days. Positive colonies were obtained in 149 specimens using M-ECC, though six of them did not contain CPE following further examination (Additional file 2: Table S2). Five of the six false-positive samples whose MIC against meropenem was more than 0.25 μg/mL could be grown in M-ECC medium. One sample contained susceptible *Enterobacteriaceae* alone, which might be grown together with carbapenem resistant non-fermenting gram-negative rods such as *Pseudomonas aeruginosa*. In contrast, only 20 specimens gave positive colonies when chromID CARBA was used; all contained CPE. M-ECC medium provided significantly higher sensitivity (99.3%) than chromID CARBA (13.9%) (Table 2).

**Table 2:** Sensitivity and specificity of M-ECC and chromID CARBA to detect *bla*_IMP_-positive CPE in stool specimens

Method		*bla*_IMP_		Sensitivity (%) (95% CI)	Specificity (%) (95% CI)	PPV (%) (95% CI)	NPV (%) (95% CI)	PLR
		Positive (*n* = 144)	Negative (*n* = 891)					
M-ECC	Positive	143	6	99.3 (96.2–100.0)	99.3 (98.5–99.8)	96.0 (91.4–98.5)	99.9 (99.3–100.0)	147.5
	Negative	1	885					
ChromID CARBA	Positive	20	0	13.9 (8.7–20.6)	100.0 (99.6–100.0)	100.0 (83.2–100.0)	87.8 (85.6–89.7)	NA
	Negative	124	891					

*CI* confidential interval, *PPV* positive predictive value, *NPV* negative predictive value, *PLR* positive likelihood ratio, *NA* not applicable

Removing cloxacillin may contribute to high sensitivity of M-ECC medium. Meanwhile, it did not contain inhibitors preventing the overexpression of AmpC and efflux pumps expressed by bacteria and non-carbapenemase producing CRE can grow in M-ECC medium [20, 21]. Therefore, further identification such as PCR was necessary to confirm isolates as CPE.

This study contains several limitations. First, we examined M-ECC medium with stool specimens containing IMP-positive CPE alone because other types are rarely found in Japan. Secondly, the amount of clinical specimens directly applied in each assay was not perfectly identical because the stool specimens were not homogeneous, and the inoculated specimens were not similar even though we used the same swab. Finally, we did not sufficiently evaluate M-ECC medium with non-CPEs and it may show lower specificity. However, in order to obtain accurate epidemiology, sensitivity is more important, and M-ECC medium should be the most suitable for CPE screening in this study.

## Conclusions

Our results indicated the difficulty in detecting IMP-type CPE with available selective media. However, we successfully developed a more selective medium, M-ECC medium, for IMP-type CPE, which would be useful in IMP-type CPE prevalent regions like Japan.

## Additional files

Additional file 1: Table S1. MIC of meropenem reduction in bacterial isolates when cultured with 250 μg/mL of cloxacillin. (DOCX 37 kb)

Additional file 2: Table S2. Characteristics of bacterial species detected in stool specimens via M-ECC and chromID CARBA. (DOCX 31 kb)

## Abbreviations

CPE: Carbapenemase-producing *Enterobacteriaceae*
