# Supplementary material for: Development of selective medium for IMP-type carbapenemase-producing Enterobacteriaceae in stool specimens
Source: BMC Infect Dis. 2017 Mar 24;17:229. doi: 10.1186/s12879-017-2312-1 (PMC5366124; doi:10.1186/s12879-017-2312-1)
Supplement: Supplementary file 1 — MIC of meropenem reduction in bacterial isolates when cultured with 250 μg/mL of cloxacillin. (DOCX 37 kb) [file 12879_2017_2312_MOESM1_ESM.docx]

**Table S1. MIC of meropenem reduction in bacterial isolates when cultured with 250 µg/mL of cloxacillin**

| Strain | Species | Type of β-lactamase | |  | MIC of MEPM (mg/L) | | |
| --- | --- | --- | --- | --- | --- | --- | --- |
|  |  | Carbapenemase | ESBL |  | Nothing added | with 50 µg/mL of cloxacillin | with 250 µg/mL of cloxacillin |
| 1504-16 | *Escherichia coli* | IMP-6 | CTX-M-15, CTX-M-2 |  | 8 | 1.5 | 1 |
| 1502-08 | *E. coli* | IMP-6 | - |  | 8 | 2 | NG |
| 1405-06 | *E. coli* | IMP-6 | CTX-M-15, CTX-M-2 |  | 3 | 2 | 1.5 |
| 1502-25 | *E. coli* | IMP-6 | CTX-M-2 |  | 3 | 1 | 0.75 |
| 1409-10 | *Klebsiella pneumoniae* | IMP-6 | CTX-M-2 |  | 1 | 0.5 | 0.5 |
| 1502-02 | *K. pneumoniae* | IMP-6 | - |  | 4 | 3 | 1.5 |
| 1409-09 | *K. pneumoniae* | IMP-6 | CTX-M-2 |  | 0.5 | 0.38 | 0.38 |
| 1212-13 | *K. pneumoniae* | IMP-6 | CTX-M-2 |  | 0.5 | 0.5 | 0.5 |
| 1412-36 | *K. pneumoniae* | IMP-1 | CTX-M-2 |  | 3 | 1 | 0.5 |
| 1502-06 | *K. pneumoniae* | IMP-1 | - |  | 1 | 1 | 1 |
| 1011-12 | *K. pneumoniae* | KPC-2 | - |  | >32 | >32 | >32 |
| BAA-1705 | *K. pneumoniae* | KPC-2 | - |  | 32 | 16 | 2 |
| BAA-2470 | *K. pneumoniae* | NDM-1 | CTX-M-15 |  | 16 | 8 | 6 |
| BAA-2471 | *E. coli* | NDM-1 | CTX-M-15 |  | >32 | >32 | >32 |
| TRKP-54 | *K. pneumoniae* | OXA-48 | - |  | 16 | 4 | 4 |
| 1502-04 | *Enterobacter cloacae* | - | - |  | 2 | 0.125 | 0.032 |
| 1012-01 | *Serratia marcescens* | - | - |  | 6 | 1.5 | 0.38 |
| 1104-18 | *E. cloacae* | - | - |  | 0.19 | 0.047 | 0.006 |
| 1004-112 | *E. coli* | - | CTX-M-2 |  | 0.016 | 0.008 | 0.008 |
| 1004-25 | *K. pneumoniae* | - | CTX-M-2 |  | 0.032 | 0.023 | 0.023 |

ESBL, extended-spectrum β-lactamase; MEPM, meropenem; NG, no growth.

Each isolate was cultured on Mueller-Hinton agar (MHA) (BD Diagnostic Systems) containing cloxacillin and MIC of meropenem was determined using Etest strips (bioMérieux Clinical Diagnostics, Marcy l’Etoile, France).
