# Supplementary material for: Development of selective medium for IMP-type carbapenemase-producing Enterobacteriaceae in stool specimens
Source: BMC Infect Dis. 2017 Mar 24;17:229. doi: 10.1186/s12879-017-2312-1 (PMC5366124; doi:10.1186/s12879-017-2312-1)
Supplement: Supplementary file 2 — Characteristics of bacterial species detected in stool specimens via M-ECC and chromID CARBA. (DOCX 31 kb) [file 12879_2017_2312_MOESM2_ESM.docx]

**Table S2. Characteristics of bacterial species detected in stool specimens via M-ECC and chromID CARBA**

| Method | Number of samples containing each isolate | | | | | | |
| --- | --- | --- | --- | --- | --- | --- | --- |
|  | CRE | | | |  | Others | |
|  | CPE  (*bla*_IMP-1_)  (n = 1) | CPE  (*bla*_IMP-6_)  (n = 142) | CPE  (Others)  (n = 1) | CRE (Non-CPE)  (n = 3) |  | Susceptible *Enterobacteriaceae*  (n = 2) | Other species  (n = 1) |
| M-ECC  (n = 149) | 1 | 142 | 0 | 3^*^ |  | 2^†^ | 1^‡^ |
| chromID CARBA  (n = 20) | 0 | 19 | 1^§^ | 0 |  | 0 | 0 |

^*^ *E. coli* (MIC; IPM 1.5 µg/mL, MEPM 3 µg/mL), *K. pneumoniae* (MIC; IPM 0.38 µg/mL, MEPM 2 µg/mL), *K. pneumoniae* (MIC; IPM 4 µg/mL, MEPM 12 µg/mL)

^†^*E. coli* (MIC; IPM 0.25 µg/mL, MEPM 0.38 µg/mL)*, Citrobacter freundii* (MIC; IPM 0.38 µg/mL, MEPM 0.19 µg/mL)

^‡^ *Stenotrophomonas maltophilia* (MIC; IPM >32 µg/mL, MEPM >32 µg/mL)

^§^ *Providencia rettgeri* (MIC; IPM > >32 µg/mL, MEPM >32 µg/mL, *bla*_IMP-10_-like)
